# A comparative analysis of the effects of green blended activators on the durability and mechanical performance of slag-based geopolymer cement

**DOI:** 10.1038/s41598-026-44669-0

**Published:** 2026-04-20

**Authors:** Fayza S. Hashem, Osama. Fadel, Hassan Soltan Hassan, Faten A. Selim

**Affiliations:** 1 Chemistry Department, Faculty of Science, Ain Shams University, Cairo, Egypt; 2 Laboratories Department, The Arab Contractors (Osman Ahmed Osman & Co.), Cairo, Egypt; 3 Geology Department, Faculty of Science, New Valley University, El- Kharga, 72511 New Valley Egypt

**Keywords:** Geopolymer, Slag, Na_2_CO_3_ activator, CaCO_3_ activator, Mixed activator, Chemistry, Engineering, Environmental sciences, Materials science

## Abstract

Investigating environmentally suitable alternatives becomes crucial as demand for Portland cement is expected to increase dramatically over the next several years. Alkaline-activated binders produced by alkali activation of aluminosilicate precursors serve as greener and viable substitutes for PC. However, conventional activators such as sodium silicate (SS) or sodium hydroxide (NaOH) are corrosive, costly, and pose significant environmental concerns. In contrast, sodium carbonate (Na_2_CO_3_, NC) and calcium carbonate (CaCO_3_, CC) are less corrosive, more affordable, and naturally abundant, making them sustainable alkali activators for AAs. To advance the use of sustainable binders, this study investigated the properties of slag activated with a blended activator composed of Na_2_SiO_3_/Na_2_CO_3_ or Na_2_SiO_3_/CaCO_3_ at room temperature. The findings reveal that using a combined activator of SS and CC reduces compressive strength and delays the setting process, whereas a blend of SS and NC improves strength by 10 to 12% over a curing period of 3–90 days. Additionally, using a blend of 7% SS + 3% NC as an activator reduces total porosity and water absorption and improves the highest bulk density. Under 5% MgSO₄ attack for six months, CC-containing mixes deteriorated severely (strength decreased to 3.1–15.4 MPa), whereas NC-activated mixes retained higher strength (34.4–40.6 MPa). After firing at 300, 600, and 800 °C, M5 retained approximately 70%, 51%, and 39% of its original 28-day strength, respectively, outperforming CC-based systems (32–84% loss). XRD, FTIR, and SEM analyses confirmed denser C–A–S–H and/or N–A–S–H gel formation in SS/NC mixes. The findings demonstrate that Na₂CO₃ is a technically viable, environmentally safer, and economically attractive partial substitute for sodium silicate, delivering enhanced durability and thermal resistance for sustainable structural applications.

## Introduction

Finding environmentally friendly substitutes is crucial because the production of Portland cement (PC), the main binder in conventional cementitious materials, accounts for about 5–10% of global anthropogenic carbon dioxide emissions^1–4^. The demand for PC is expected to increase dramatically over the next several years^5,6^. Many studies have used Supplementary cementitious materials (SCMs) such as fly ash, silica fume, and metakaolin to improve PC properties, but the environmental impacts associated with its manufacture remain a crucial problem^7,8^. In recent years, the use of geopolymers as binders in place of conventional Portland cement has become more common. The primary drivers of this interest are the high carbon dioxide emissions from the production of Portland cement and the shortage of raw materials, particularly limestone. Geopolymers are created by alkali activation of aluminosilicate minerals, which can come from natural sources like kaolin and bentonite^9,10^. Alternatively, it can be achieved using industrial wastes and by-products, including fly ash and slag. Depending on the raw materials used in their manufacturing, geopolymers have a variety of properties. Their main benefits include their quick-strength development, recognized resistance to heat action and corrosive ions, remarkable chemical resistance, and capacity to immobilize toxic and hazardous wastes^11^.

Geopolymers are typically made by combining liquid alkaline activator solutions with aluminosilicate binders. Unfortunately, the corrosive and viscous nature of these activation solutions restricts and complicates the use of geopolymers in construction^12^. The term “one-part geopolymer” (OPG) refers to a method that replaces traditional liquid alkaline solutions with solid alkaline activators that are pre-mixed with binder powders. In this process, adding water to the mixture resembles the conventional process used in cement production^13^. Any substance that provides alkali or alkaline cations, elevates the pH of the reaction mixture, and facilitates dissolution can be used as the alkali source, also known as an activator, in a one-part geopolymer mix^14^. The typical alkaline activators used are NaOH, Na_2_SiO_3,_ or a combination of the two. Numerous authors have demonstrated that a slag mixture activated with sodium silicate attains the highest compressive strength among alkali-activated materials and offers superior durability^15,16^. However, slag activated with sodium silicate tends to harden more quickly, resulting in increased shrinkage. This tendency poses challenges for its implementation in construction projects^17^. However, in large-scale industrial facilities, activating slag with sodium hydroxide usually requires a high activator concentration, raising significant safety and occupational health concerns. Furthermore, the use of highly alkaline activators during mixing and the need to handle the resulting cement carefully limit the long-term use of geopolymer^18^. Regarding occupational health and safety, NaOH and concentrated sodium silicate solutions are highly alkaline (pH > 13), making them strongly corrosive. Large-scale industrial handling requires specialized storage systems, protective equipment, and strict safety procedures because of the risks of chemical burns, eye damage, and respiratory irritation. Luukkonen et al. emphasized that the corrosive nature and handling risks of traditional alkaline solutions limit the widespread adoption of AAM technology in practical construction applications^19^. Even at the laboratory scale, preparing NaOH solutions is highly exothermic and requires fume hoods, face shields, and chemical-resistant gloves to prevent injuries.

Numerous studies have explored the use of alternative alkaline activators, such as magnesium oxide (MgO) and calcium oxide (CaO), in fly ash-based geopolymers^20^ while CaO, Ca(OH)_2_, MgO were used in slag systems^20,21^. Abdel-Gawwad et al. used dolomite and MgO to activate slag systems for the formation of GP^22^. Finding more environmentally friendly substitutes for potent alkaline sources has been the subject of numerous investigations recently. One such method is the production of activators from industrial and agricultural waste by employing calcium-rich lime and nearly neutral sodium sulfate and carbonate salts^23,24^.

Na_2_CO_3_ (NC) powder is an easier and safer material to handle, and its production process is more environmentally friendly. However, the dissolution of Na_2_CO_3_ creates a buffered alkaline environment in the aqueous phase^25,26^. In the Na_2_CO_3_-activated granulated blast furnace slag, the initial pH is below 12, and the alkalinity increases slowly during the alkali-activation process^27^. The reaction between Ca^2+^ dissolved from slag and CO_3_^2-^ released from Na_2_CO_3_ leads to precipitation of CaCO_3_. In comparison to slag binders activated using Na₂SiO₃-activated or NaOH-activated slag geopolymer, those activated with Na₂CO₃ often exhibit slower strength development and delayed hardening because the alkalinity can rise sufficiently to initiate the later stages of alkali activation^28,29^. The literature claims that alkali-activated slag-based (AAS) cement made with Na_2_CO_3_ exhibits a gradual increase in strength in the early phases and a notable one in the later phases. The AAS mortar that Li and Sun created, activated with Na₂CO₃, was plastic after three days but reached a strength of 60 MPa after twenty-eight^30^.

Recently, numerous studies have investigated the impact of using CaCO_3_ (calcium carbonate) or limestone minerals on the properties of alkali-activated slag (AAS) or traditional PC mix^31^. According to Perez-Cortes et al., adding CaCO_3_ to the geopolymer mix provides several advantages. Notably, it can decrease the amount of alkaline activator needed to activate the geopolymer mix, while also improving workability^32^. Additionally, CaCO_3_ provides extra Ca^2+^ ions, which enhance the formation of Ca-containing(i.e., N-(C)-A-S-H) gels that have a three-dimensional network structure. Including these gels, e.g. C-S-H gel, that can lead to denser microstructures and higher compressive strengths^32–35^. Dombrowski et al.^36^ examined the impact of calcium content on fly ash-based geopolymer and concluded that high calcium content in raw precursors results in the formation of significant amounts of C-S-H. Columbu et al. reported in their study that the fine particles of CaCO_3_ can provide additional nucleation sites for precipitates during the alkali-activation process^37,38^. Although the use of fine CaCO_3_ in geopolymer cement has been the focus of many studies, the scientific understanding of pure CaCO_3_ as an alkaline activator, whether used alone or in combination with other activators like sodium silicate and sodium carbonate, is still limited, particularly in one-part geopolymer cement formulations. More research is specifically needed to determine how it affects phase assemblages, strength development, and alkali-activation kinetics in both the early and later stages.

The primary objective of this work is to study the availability and use of greener, more cost-effective alkaline activators, such as sodium and calcium carbonates, and to compare their effectiveness with that of sodium silicates. This is done by evaluating the mechanical properties and performance of a one-part geopolymer cement made from ground granulated blast furnace slag (GGBFS) and activated with a more environmentally friendly alkaline activator, which is a mixture of two alkali activators in different molar ratios: sodium silicate (SS) and calcium carbonate (CC) or sodium silicate (SS) and sodium carbonate (NC). The geopolymer’s physicochemical characteristics and compressive strength are evaluated up to 28 days after hydration. The geopolymer’s resilience to two harsh environments is also evaluated: exposure to temperatures as high as 800 °C and six months of exposure to sulfate ions.

## Experimental

### Materials

**Fig. 1:**
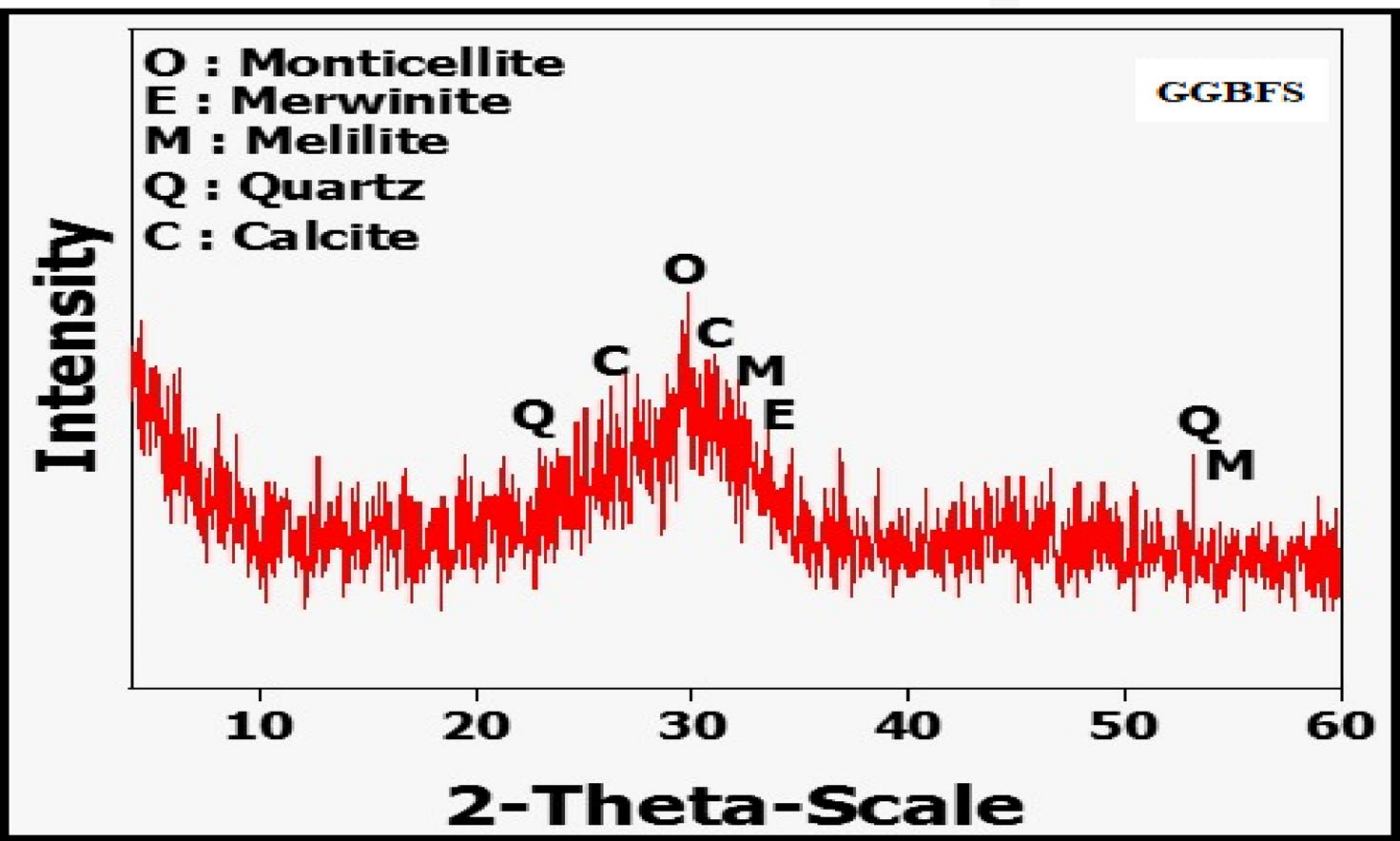
XRD of ground granulated blast furnace slag.

The following materials were employed in this study:

- Ground granulated blast furnace slag, or BFS, is a byproduct of pig iron production. The GGBFS used in this study came from a steel foundry in the Egyptian province of Helwan and has a Blaine-specific surface area of 2883 cm²/kg. Figure 1 shows the results of the X-ray diffraction (XRD) study, and Table 1 provides a detailed description of its chemical makeup.
- Sodium silicate (Na_2_SiO_3_.9H_2_O, SS), calcium carbonate (CaCO_3,_ CC) and sodium carbonate (Na_2_CO_3,_ NC) were used as alkaline activators for the fabricated geopolymer (GP). These chemicals (with a purity ≥ 98%) were provided by Alfa Aromatic Company.

### Geopolymer synthesis

Geopolymer mixes were prepared using Ground Granulated Blast Furnace Slag (BFS) combined with various ratios of alkaline solid activators, as outlined in Table 2. In this work, we select 10% Na₂SiO₃ (by weight of slag) as the control mix was based on both literature evidence and preliminary optimization considerations^39,40^. The dry ingredients, including solid activators such as SS or mixes of SS with CC or NC, were thoroughly mixed into a homogeneous slurry using a ball mill for six hours to create the one-part geopolymer. The appropriate amount of water was then added to produce a variety of geopolymer pastes with a standard consistency to ensure all the tested pastes have the same workability. The resulting pastes were molded into one-inch cubic specimens (2.54 cm^3^) using cubic molds. The molds were compacted with vibrations, and the paste surfaces were leveled and smoothed with a thin-edged trowel. After casting, the specimens were demolded and cured at humidity chamber at a temperature = 25 ± 2 °C for 3, 7, 28, and 90 days.

**Table 1:** The chemical oxide compositions of GGBFS, wt %.

Oxide %	GGBFS
SiO_2_	38.9
Al_2_O_3_	10.66
Fe_2_O_3_	1.20
CaO	38.51
MgO	4.39
SO_3_	1.96
K_2_O	0.49
Na_2_O	0.91
Cl	0.04
L.O.I	2.94

**Table 2:** The percentage composition of the different geopolymer mixes and their designations.

Mixes	Slag (kg/m^3^)	Na_2_SiO_3_ (kg/m^3^)	CaCO_3_ (kg/m^3^)	Na_2_CO_3_ (kg/m^3^)
M1	100	10	–	–
M2	100	7	3	–
M3	100	5	5	–
M4	100	3	7	–
M5	100	7	–	3
M6	100	5	–	5
M7	100	3	–	7%

### Testing and characterization

The ASTM C191 standard test was used to evaluate the setting times of the manufactured GP mixes^41^. The test involved measuring the initial and final setting times for each of the freshly made geopolymer pastes using the Vicat needle instrument. This device consists of a frame with a movable rod that has a needle that may be fastened to one end and a disc on the other. Following the standard test of ASTM C109 M16-a^42^, the compressive strength test was conducted on three cubic samples for each geopolymer mix after curing for 3, 14, 28, and 90 days. To evaluate the compressive strength data, a Ton Industrie-machine (West Germany) with a maximum capacity load of 60 tons was used.

### Durability tests

- The specimens of GPC have been tested for resistance to deterioration from firing and sulfate ion attack. Cubic specimens are cured for 28 days, and then immersed in a 5% magnesium sulfate (MgSO_4_) solution for 1, 3, and 6 months to study the sulfate ion attack. Throughout this immersion period, the MgSO_4_ solution is periodically replaced. Following each period of one, three, and six months. The specimens undergo a test for compressive strength^43^.
- Firing resistance is assessed by exposing the 28-day cured GPC specimens at temperatures of 300 °, 600 °, and 800 °C for three hours in a muffle furnace by heating rate 10 degree/minute. After firing the specimens, the heated cubic samples were gradually cooled to room temperature in desiccators, and the samples were subjected to the compressive strength test^13,42^.

**Fig. 2:**
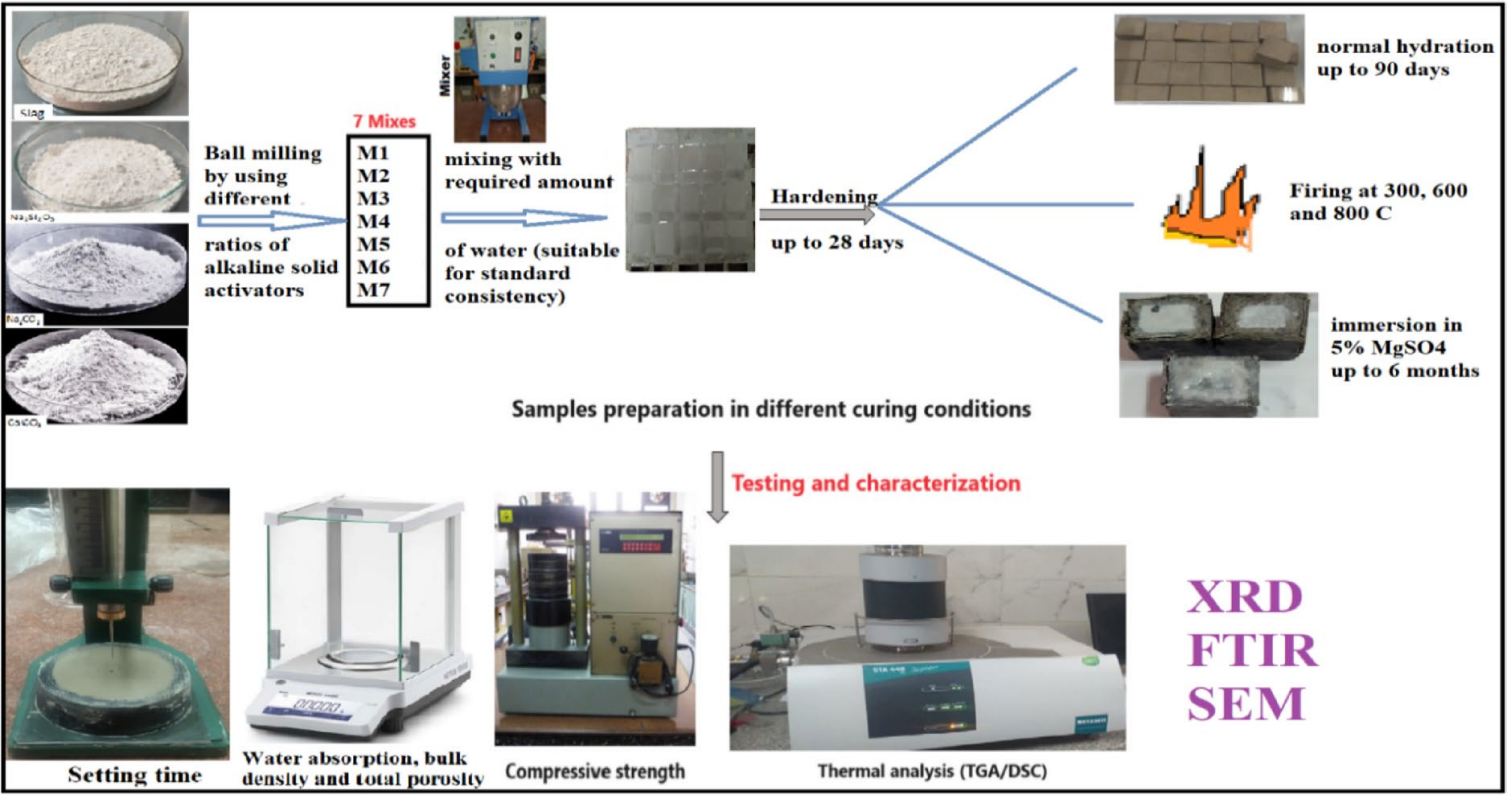
Schematic diagram to summarize the experimental work.

Selected hydrated and dried geopolymer specimens were subjected to X-ray diffraction to investigate their mineral composition and crystalline structure. Additionally, the functional groups in the geopolymer composite (GP) were identified using Fourier Transform Infrared (FTIR) spectroscopy on specific hardened geopolymer samples. Spectral bands were recorded between 4000 and 400 cm^− 1^. The shape and microstructure of a few chosen hardened GPC specimens were also examined using a scanning electron microscope (SEM). Figure 2 shows a schematic diagram of the summary of the experimental work done in this study.

## Results and discussion

### Setting time and water consistency

**Fig. 3:**
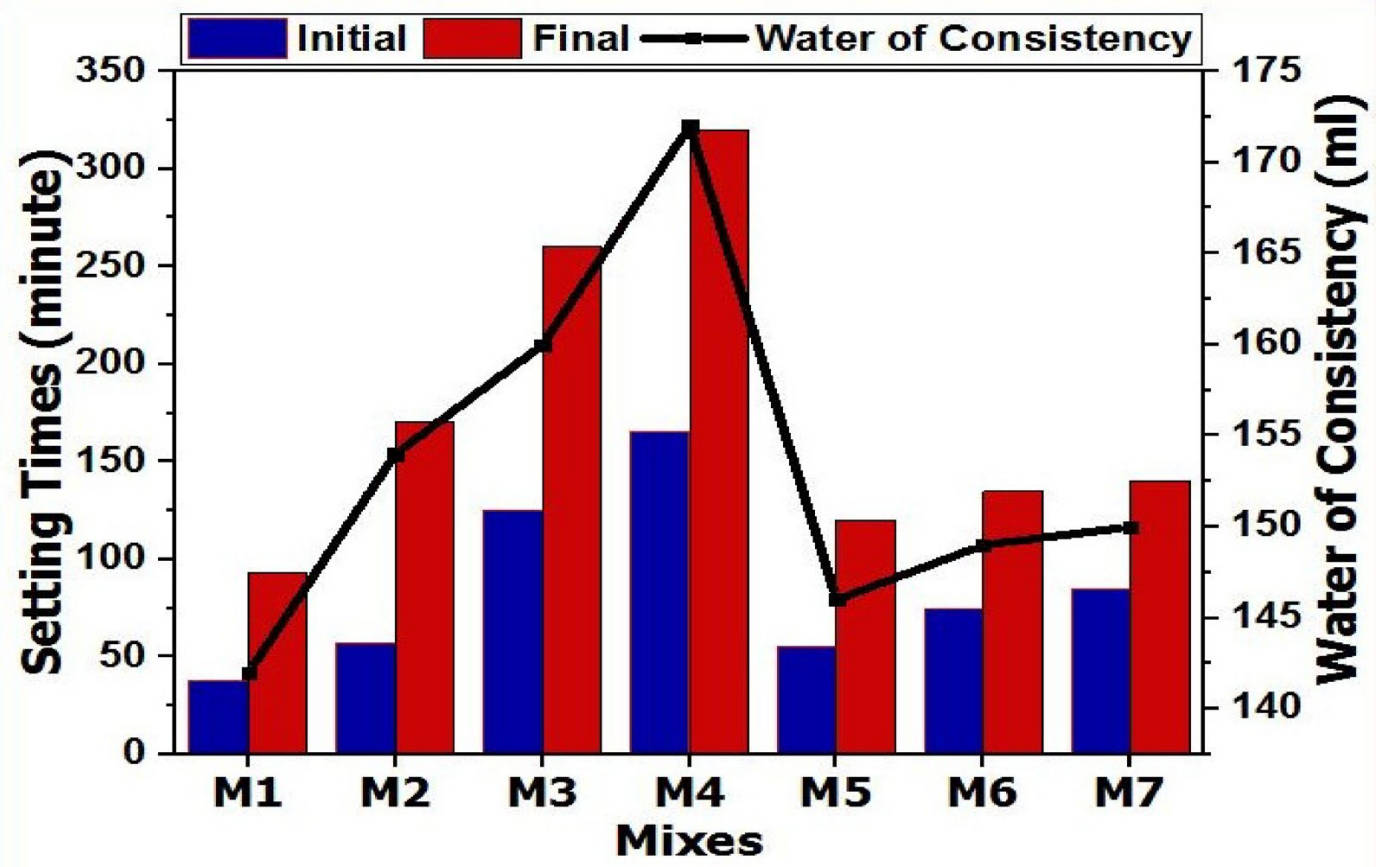
Setting times and water consistency for various GP mixes.

One important factor that affects how well building materials are used is the setting time. The alkalinity of the activator and the reactivity of the precursor have a major impact on the setting process in geopolymer cement. Figure 3 illustrates the initial and final setting times, as well as the standard water consistency of activated slag-based geopolymer pastes activated by various activator mixes. The initial setting times for all the fabricated mixes ranged from 38 to 165 min, while the final setting times varied from 93 to 320 min^44^. The geopolymer-based slag activated with 10% sodium silicate (Na_2_SiO_3_), labeled as (M1), showed a fast-setting process, and it also had the lowest standard water consistency among the mixes tested. The quick setting of the M1 mix is likely due to the heat generated during dissolution of the solid activator.

According to previous studies, the stiffness of the geopolymer paste is also accelerated by an exothermic process that occurs when the activator and slag particles combine^39,45^. However, geopolymer mixes activated by a blend of SS/NC (M5-M7) showed shorter setting times compared to those activated by a blend of SS/NC (M2-M4). The high miscibility of NC rather than CC is responsible for such a result. Besides, the formal mixes, those active by SS/NC, showed low water consistency compared to the latter mixes, active by SS/CC. Another explanation can be added for delaying the setting process in mixes M2-M4, that when the water consistency increases in the GP mixes, the water consumption increases, which slows down the setting process by absorbing some of the heat generated during alkali activation. The higher water demand in mixes M2-M4 is expected due to lower activator solubility and slower reaction kinetics. Compared with typical slag-based AAMs reported in literature (water/binder ratios (0.30–0.40), the obtained values fall within the normal range^46,47^. Besides, the GP matrix was diluted as a result of the higher water need in GP mixes, which lowers pH^46^. Shi and Day claim that the activator solution’s initial pH is crucial in controlling the GP mix’s early reactions; as a result, a drop in pH could cause the setting time to be further delayed^47^. Furthermore, the low alkalinity (pH level) of the alkaline activator may promote the release of carbonate ions (CO_3_²⁻) in the geopolymer (GP) mix when the ratio of calcium carbonate (CC) to sodium carbonate (NC) in the activator mix is high. Because of this, sodium-calcium carbonate rather than calcium silicate hydrate (C-S-H) gel is formed, which is what gives the initial stiffness of the geopolymer paste^19,48,49^.

### Compressive strength

**Fig. 4:**
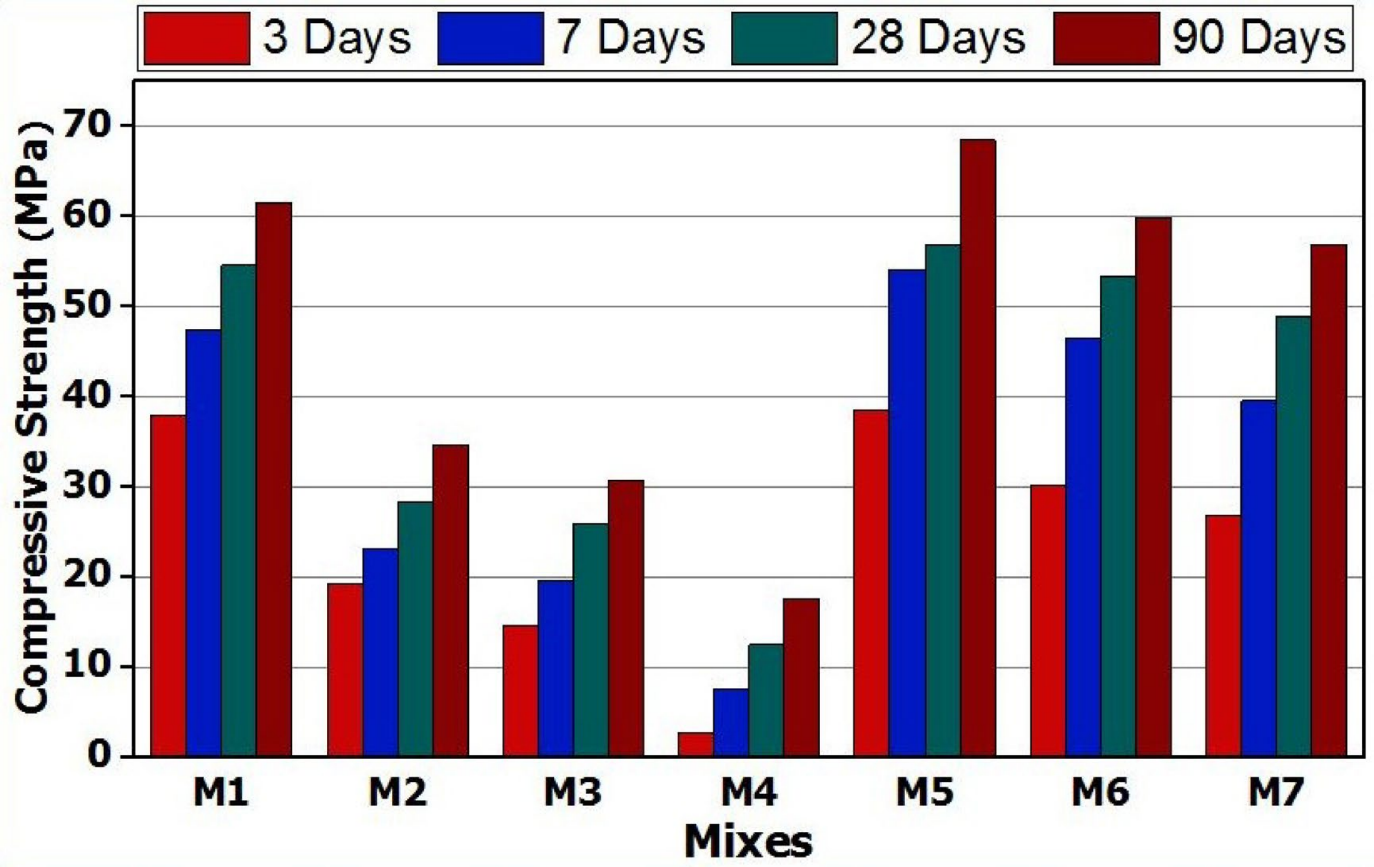
Compressive strength versus curing times for various GP mixes.

The compressive strength values for slag-based geopolymer cement prepared using 10% Na_2_SiO_3_ (SS) as an alkaline activator (M1) and those activated with blended activators made from SS/CC or SS/NC (mixes M2-M4 and M5-M7, respectively) after 3, 7, 28, and 90 days of curing are shown in Fig. 4. All the investigated mixtures’ compressive strength values showed a steady rise with curing age, up to 90 days. The alkali activation of blast furnace slag (BFS) results in the development of geopolymer gel with improved mechanical characteristics, as evidenced by the observed increase in compressive strength. Slag activation encourages the Ca-O, Si-O, and Al-O links in its structure to break, creating dissolved species as;{Ca^2+^, [H_2_SiO_4_]^2−^, [H_3_SiO_4_]^−^ and [Al(OH)_4_]^−^}, which can precipitate forming C-S-H gel and C-A-S-H hydrate^50^. According to Yip et al., the calcium ions found in blast furnace slag (BFS) balance the charge of aluminum atoms (Al³⁺) by integrating into the Si-O-Al gel structure. The Calcium-Alumino-Silicate-Hydrate (C-A-S-H) system and the Sodium-Alumino-Silicate-Hydrate (N-A-S-H) gel are both made possible by this integration. Consequently, a denser structure is created^51,52^.

The geopolymer cement (GP) specimens made with a blended activator of sodium silicate (SS) and calcium carbonate (CC) exhibited the lowest compressive strength across all tested ages compared with other mixtures. Notably, the M2 mix, which used a blend of 7% sodium silicate and 3% calcium carbonate, showed a reduction of approximately 45% in compressive strength after 90 days compared with the M1 mix, which was prepared using 10% sodium silicate. Additionally, after the same curing period, the M4 mix—which was made with 3% sodium silicate and 7% calcium carbonate—showed a notable 70% drop in compressive strength. The low miscibility of CC compared with SS lowers the initial pH of the geopolymer mix, which hampers the miscibility of Ca^+ 2^, Si^+ 2^, and Al^+ 3^ ions. This substantial decrease in matrix alkalinity, brought on by replacing sodium silicate (SS) with calcium carbonate (CC), is responsible for the strength loss seen in GP mixes (M2–M4). Hashem et al. state that the alkalinity of the alkali solution is essential to the geopolymer’s hydration process^38,52^. Wang et al. showed in their investigation that after 28 days, the pH values of the pore solution in metakaolin (MK) containing 20% calcium carbonate (CC) dropped below 10. The dissolution of geopolymer precursors is hampered by this pH reduction, which slows the rate of reaction and lowers the specified strength^53^.

GP specimens where 3% of the sodium silicate activator was replaced with sodium carbonate (M5) showed an improved compressive strength of approximately 10–12.5.5% during the 3–90 day curing period compared to M1. In contrast, mixes M6 and M7 demonstrated compressive strength values similar to M1 at the same curing times. The high miscibility of NC, compared to SS, will lead to enhancing the pH level inside the geopolymer mix. As a result, the concentrations of silicon and aluminum ions will rise in the slag mixture, which explains why using a mixed SS/NC activator results in improved mechanical properties. This procedure produced calcium-aluminum-silicate-hydrate (C-A-S-H) rapidly, primarily in the first 3-4days^54^. Some researchers believe that the improvement in mechanical properties is due to the reinforcing function and pore-filling actions of Na_2_CO_3_ crystalline particles^55^. This outcome is consistent with research by Bernal et al., who discovered that a 50:50 mixture of sodium silicate and sodium carbonate was the most effective activator for slag-based geopolymers^27^. This mixture of activators will improve the mechanical properties, enhance the microstructure, and compactness of the matrix.

### Durability tests

#### MgSO_4_ attack

**Fig. 5:**
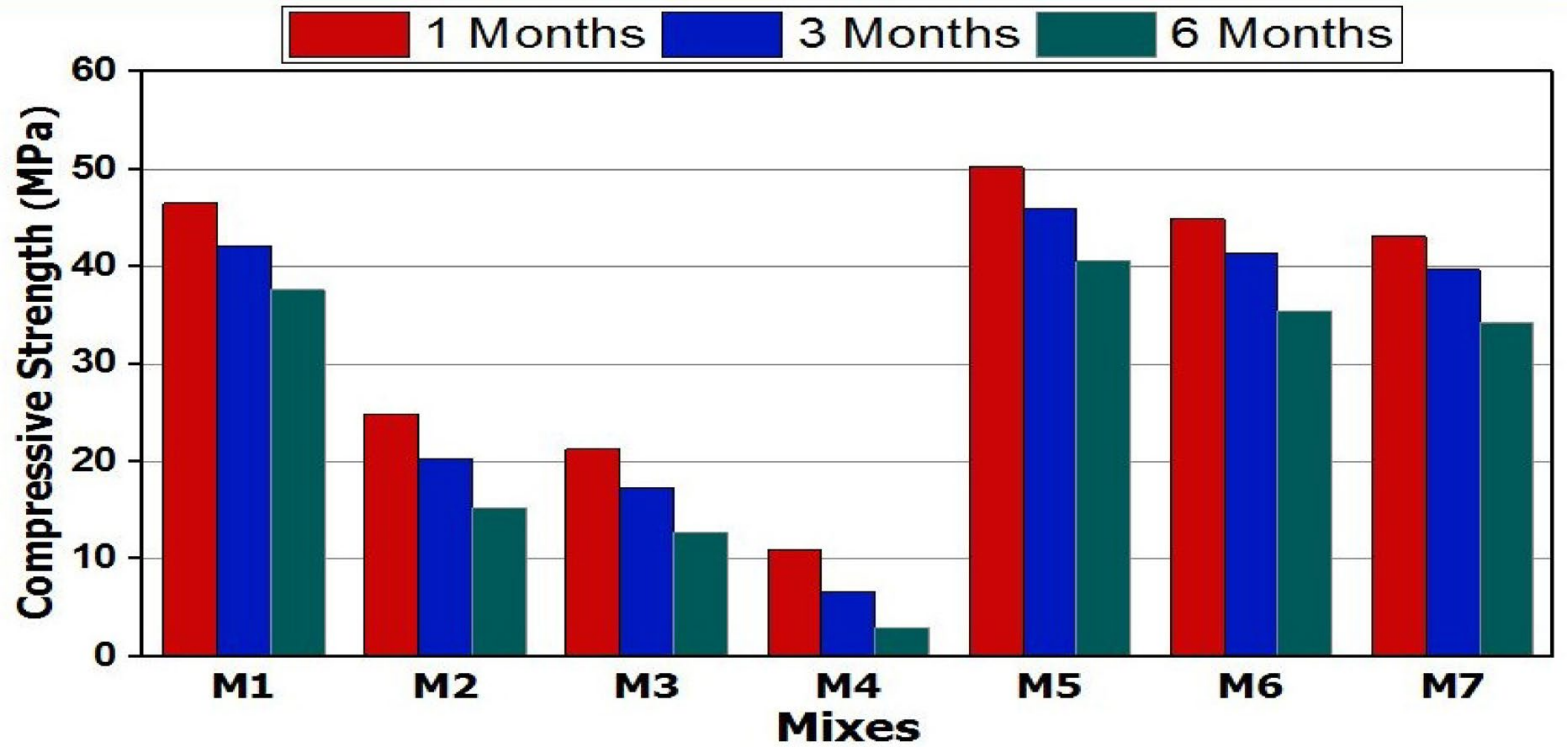
Compressive strength versus curing times for various GP mixes after immersion in MgSO_4_ solution.

The compressive strength of the GPC composites (M1–M7) following up to six months of immersion in a 5% MgSO_4_ solution is displayed in Fig. 5. All GP mixes showed a steady decline in compressive strength as the length of time immersed in magnesium sulfate solution increased. This could be explained by SO_4_^−2^ ions attacking the geopolymer mixes through a sequence of deterioration reactions that produced new phases such as gypsum and ettringite^55,56^. Gypsum and Ettringite, which are characterized by their large volumes, as well as their presence within the solidified geopolymer matrix, cause internal tension, leading to microcracks to occur and alkalis to migrate from geopolymers into the solution via the open pores^57^. Furthermore, the pH is lowered when gypsum was formed inside the GP matrix. At lower pH values, calcium alumino-silicate hydrate (CASH) becomes unstable^58^.

GPC specimens made by substituting various ratios of SS with NC activator demonstrated a comparable loss in compressive strength across all immersion times in sulfate solution. After six months of immersion, the compressive strength values for mixes M5–M7 ranged from 40.6 MPa to 34.4 MPa, In contrast, GPC specimens made by substituting sodium silicates with different percentages of calcium carbonate showed the lowest compressive strength values. After six months of immersion in sulfate solution, the compressive strength decreased from 15.4 MPa (M2) to 3.1 MPa (M4). The low resistance to attack by SO_4_^−2^ ions noticed in GPC specimens that were prepared using calcium carbonate as an activator can be related to the decalcification of the principal binding gel phases, such as C–S–H and C–A–S–H, caused by the entry of Mg²⁺ ions, leading to the formation of large amounts of Ettringite. However, when sodium carbonate is introduced as an activator in GP mixes, sodium aluminosilicate hydrates (N-A-S-H) gel is preferably created, which has a superior mechanical properties^59^.

#### Fire resistance

**Fig. 6:**
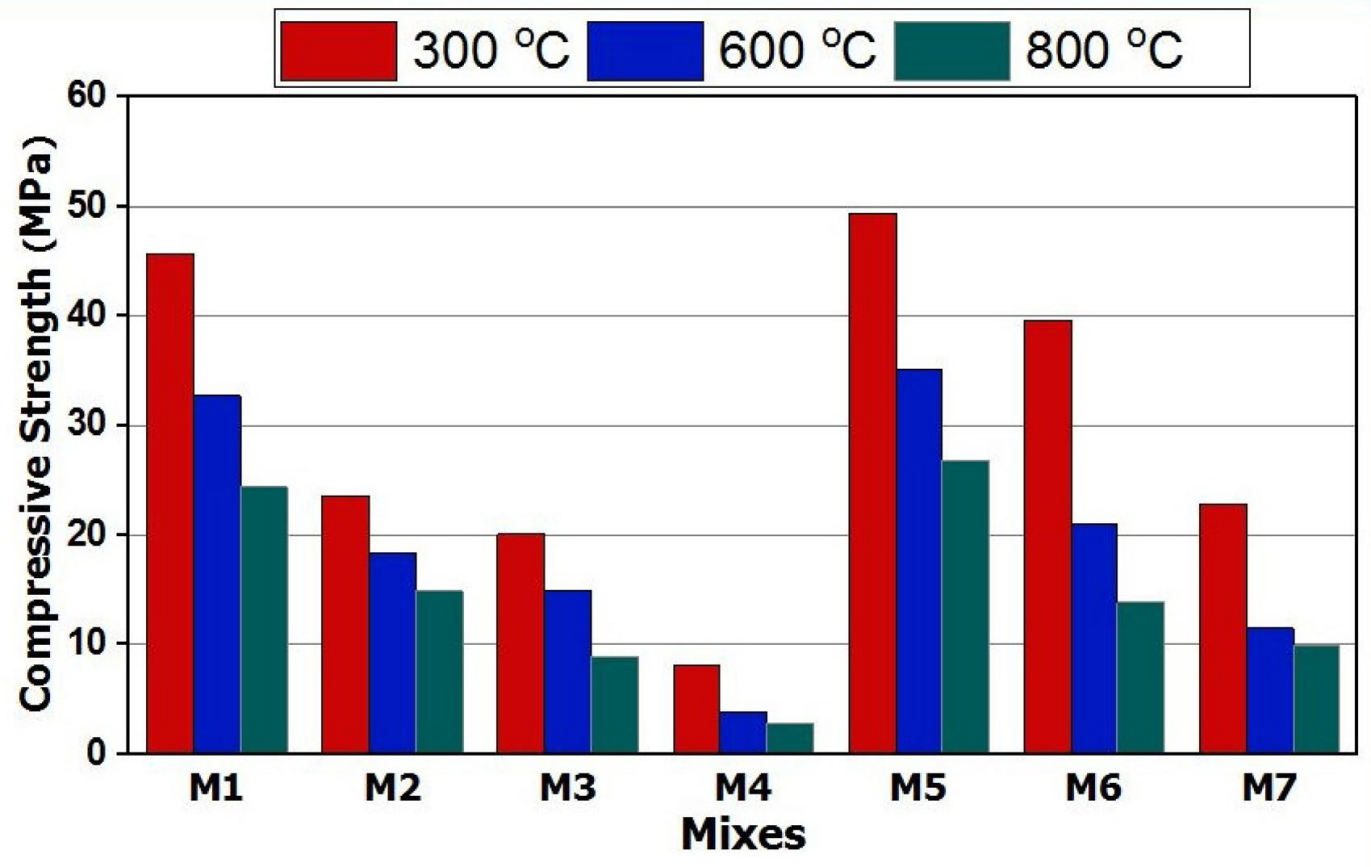
Compressive strength of GP mixes after firing at 300 °C, 600 °C and 800 °C.

The compressive strength of all GPC mixes (M1-M7) after curing in 100% RH then firing at 300 °C, 600 °C and 800 °C for 3 h in a muffle furnace at heating rate 10 degree/minute followed by slow cooling to room temperature in a closed desiccator was illustrated in Fig. 6^14,19,39^. All GPC specimens exhibited a continuous decline in compressive strength, which can be attributed to the dehydration and decalcification of the geopolymer hydration products and thermal deterioration inside the geopolymer specimens that leads to cracks formation and pores broadening followed by drops in compressive strength^59^. These results inconsistence with those of Verma et al., who explained the failure of strength of thermally treated GPC samples between 600 and 900° C to the water evaporation and dehydration of the geopolymer matrix and the melting of the bonding between the matrices^60^.

GPC specimens made by using CaCO_3_ as an activator with Na_2_SiO_3_ show a dramatic failure in the compressive strength values and the compressive strength loss ranging from 32% − 84% for mixes M2-M4 at the different firing temperatures. Such failure in strength can be related to decarbonation and dehydration process occur for the GP hydration products as well as the release of H_2_O to form larger size particles^61^. On the other hand, GPC specimens made by using Na_2_CO_3_ as activator with Na_2_SiO_3_ shows a slight boosting in CS by firing at all temperatures and the loss ranging from 30% − 82% for mixes M5-M7 for all firing temperatures, so M5 contain (7% Na_2_SiO_3_ + 3% Na_2_CO_3_), which showed the highest fire resistance among the tested mixes. They retained approximately 70%, 51%, and 39% of their 28-day strength after being fired at 300 °C, 600 °C, and 800 °C, respectively. The extent of strength loss mainly depends on the types of hydrates formed, as calcium-rich hydrates in mixes M2–M4 exhibit lower heat resistance compared to the silica-, silica/alumina-, or silica/sodium-rich hydrates found in mixes M5–M7^61,62^.

### X-ray diffraction

**Fig. 7:**
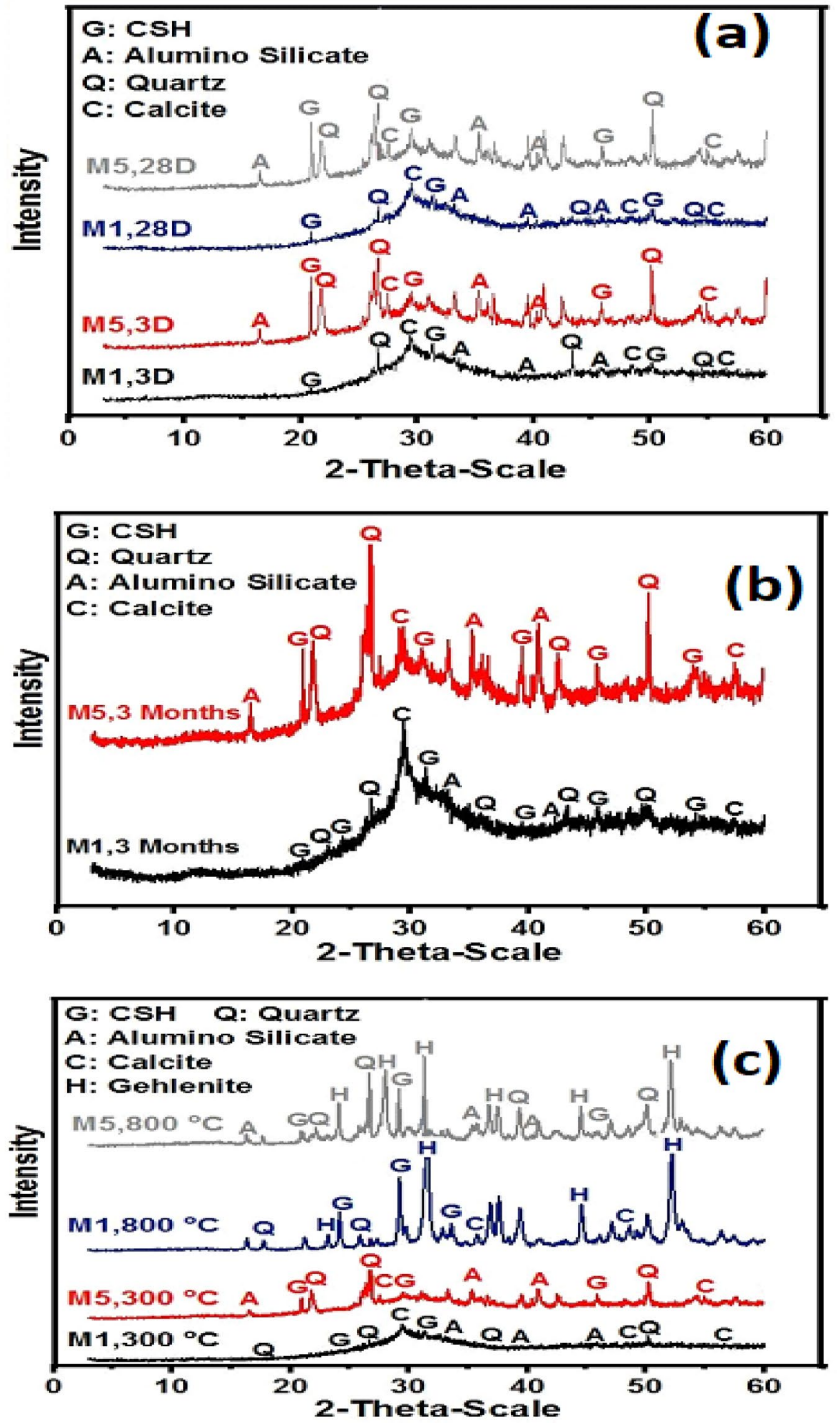
XRD patterns of mixes M1 and M5 (**a**) after 3 and 28 days of curing in 100% RH, (**b**) immersion in 5% MgSO_4_ for 3 months and (**c**) firing at 300 °C and 800 °C.

The XRD of GPC mixes activated by 10% SS and (7% SS + 3% NC), mixes M1 and M5, respectively, after 3 and 28 days, is displayed in Fig. 7a. For the two specimens studied, XRD patterns showed a wide diffraction hump between 26–29.5.5° 2θ corresponding to CSH gel phase that formed in GPC pastes^63^. Additionally, calcium alumino-silicate phases (C-A-S-H) gel, which are hydration products of GPC, are present. Besides, M5 exhibits extra peaks for sodium alumino-silicate phases (N-A-S-H) and calcium sodium silicate hydrates (N(C)-A-S-H). These phases are responsible for the stiffness and the gained compressive strength in these two mixes^14,19,39,40^. Furthermore, peaks due to unreacted quartz are also identified. After 28 days of curing, the intensities of the hydrated phase peaks increase, while the peak intensity of unreacted quartz decreases, indicating the progression of polymerization reactions.

XRD spectra of GPC specimens mixes M1 and M5 after immersion in 5% MgSO_4_ up to 3 months is shown in Fig. 7b. XRD patterns explore the same peaks of the hydration products as those found after 28 days of normal hydration. However, the intensity of C-S-H gels and C-A-S-H gels in M5 were higher than that in M1. This confirms that the compressive strength results of GPC made by using sodium carbonate activator show resistance to sulfate attack compared to GPC made by using sodium silicate activator only.

XRD patterns of SS/NC mixes showed a broader amorphous hump with limited crystalline carbonate phases, indicating the formation of a more polymerized C–(N)–A–S–H type gel with partial sodium incorporation. In contrast, SS/CC mixes exhibited stronger reflections associated with calcite and other calcium-rich phases, suggesting incomplete reaction and higher residual carbonate content. After thermal exposure, the SS/CC systems showed intensified cracking and microstructural disruption.

XRD of mixes M1 and M5 after firing at 300 °C and 800 °C for 3 h is shown in Fig. 7c. The XRD pattern of the mixes heated to 300 °C displays increased peak sharpness corresponding to C–S–H gel and aluminosilicate phases, attributed to hydrothermal reactions occurring during firing at this temperature^63^. XRD patterns of SS/NC mixes showed a broader amorphous hump with limited crystalline carbonate phases, indicating the formation of a more polymerized C–(N)–A–S–H type gel with partial sodium incorporation. Firing at 800 °C results as showed in XRD pattern shows formation of dehydrated phases like gehlenite (Ca_2_Al_2_SiO_7_) which possess low mechanical properties. These results agree with the results of compressive strength development obtained.

### Fourier transform infrared (FTIR) spectroscopy analysis

**Fig. 8:**
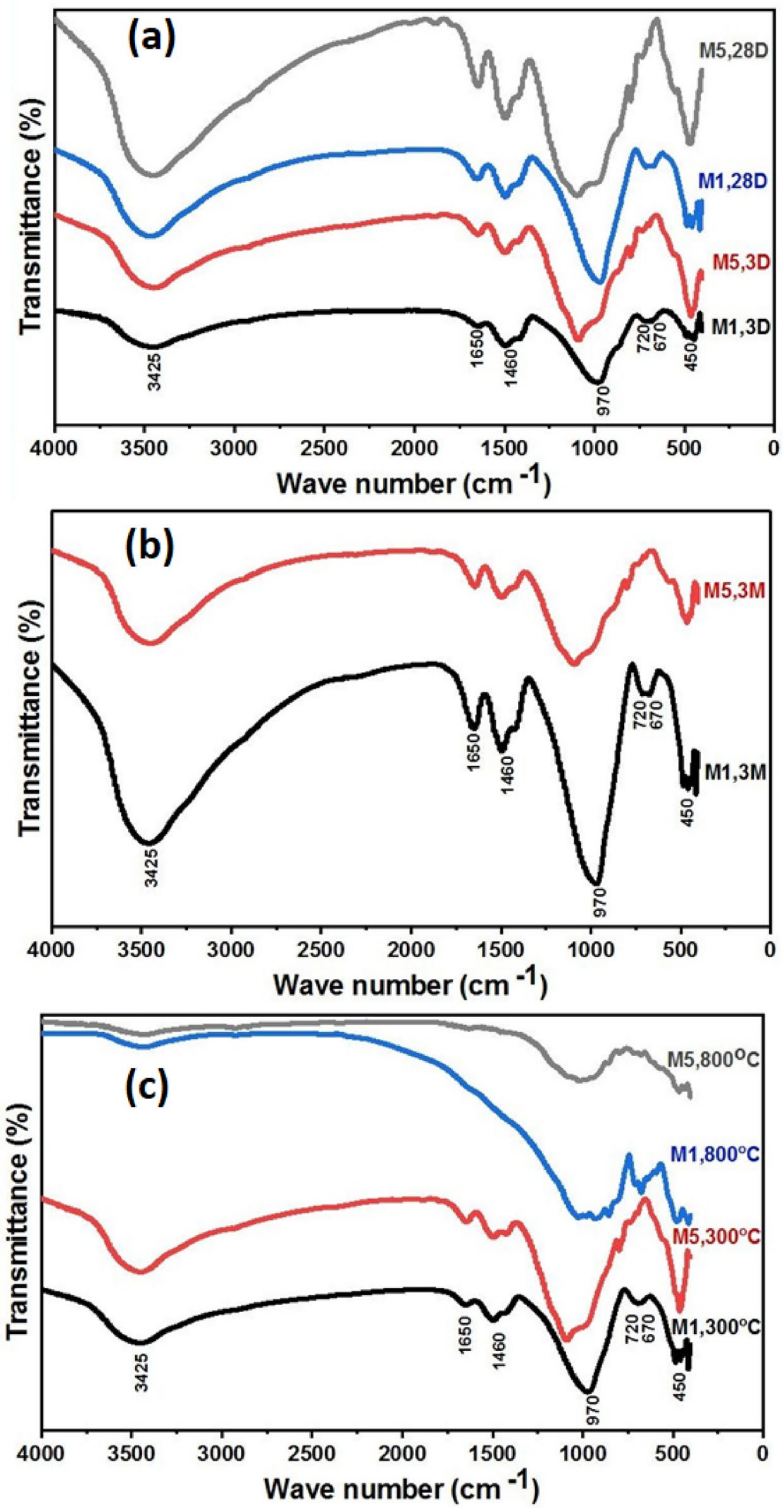
FTIR spectra of mixes M1 and M5 (**a**) after 3 and 28 days of curing in 100% RH, (**b**) immersion in 5% MgSO_4_ for 3 months and (**c**) firing at 300 °C and 800 °C.

The formation of reaction products in the pastes was further evidenced with the help of FTIR spectra as a supplement to X-ray structural analysis Fig. 8. The IR spectra of geopolymer samples (M1 and M5) were recorded after curing for 3 and 28 days under ambient conditions and 100% relative humidity. All tested GPC specimens showed broad bands around 3425 and 1650 cm-1, attributed to the bending vibration of H-O-H and the stretching vibration of OH. These absorption bands are attributed to crystalline H₂O in the hydrated products (C–S–H) and (C–A–S–H), in addition to occluded water molecules within the geopolymer network^64,65^. Vibrational stretching of O-C-O was observed for all GPC specimens at 1460 cm-1, which is due to the carbonation reaction^66^. The creation of an aluminosilicate gel is indicated by the band seen at about 970 cm⁻¹, which is ascribed to the asymmetric stretching vibrations of Al–O–Si and Si–O–Si bonds. Furthermore, the Si–O–Si symmetric stretching vibrations were found to be between 670 and 720 cm⁻¹. Additionally, O-Si-O and Si-O-Si vibrational bending was discovered at 450 cm-1^67^. FTIR spectra of M5 mixes revealed a shift of the main Si–O–Si and/or Si–O–Al bands toward lower wavenumbers, indicating higher polymerization and greater Al incorporation into the silicate network. This more cross-linked aluminosilicate framework enhances thermal stability because N–A–S–H-type structures contain stronger covalent Si–O–Al bonds and lower bound water content compared to calcium-rich hydrates. Besides, a rise in intensity of broad bands appeared in the IR spectra of these mixes, in the region of 3425 and 1650 cm^− 1^ which are assigned to stretching of (-OH) and bending (H-O-H) vibrations of bound water molecules, which are surface absorbed or entrapped in the large voids of the polymeric network^68^. This suggests that the Geopolymers’ ability to absorb water within their three-dimensional framework increases with the incorporation of Na₂CO₃ as an activator. The FTIR results align well with the compressive strength values of the samples.

The FTIR spectra of M1 and M5 after three months of immersion in 5% MgSO4 are shown in Fig. 8b. These spectra show the same stretching and bending vibrational bands, along with a notable increase in band intensity characteristic of the hydration products, suggesting that M5 has superior sulfate resistance compared to M1. Additionally, the FTIR spectra of M1 and M5 after firing at 300 and 800 °C are shown in Fig. 8c. These spectra likewise display the same bands, but M5’s band intensity is higher than M1’s, indicating that it has increased fire resistance.

### SEM

**Fig. 9:**
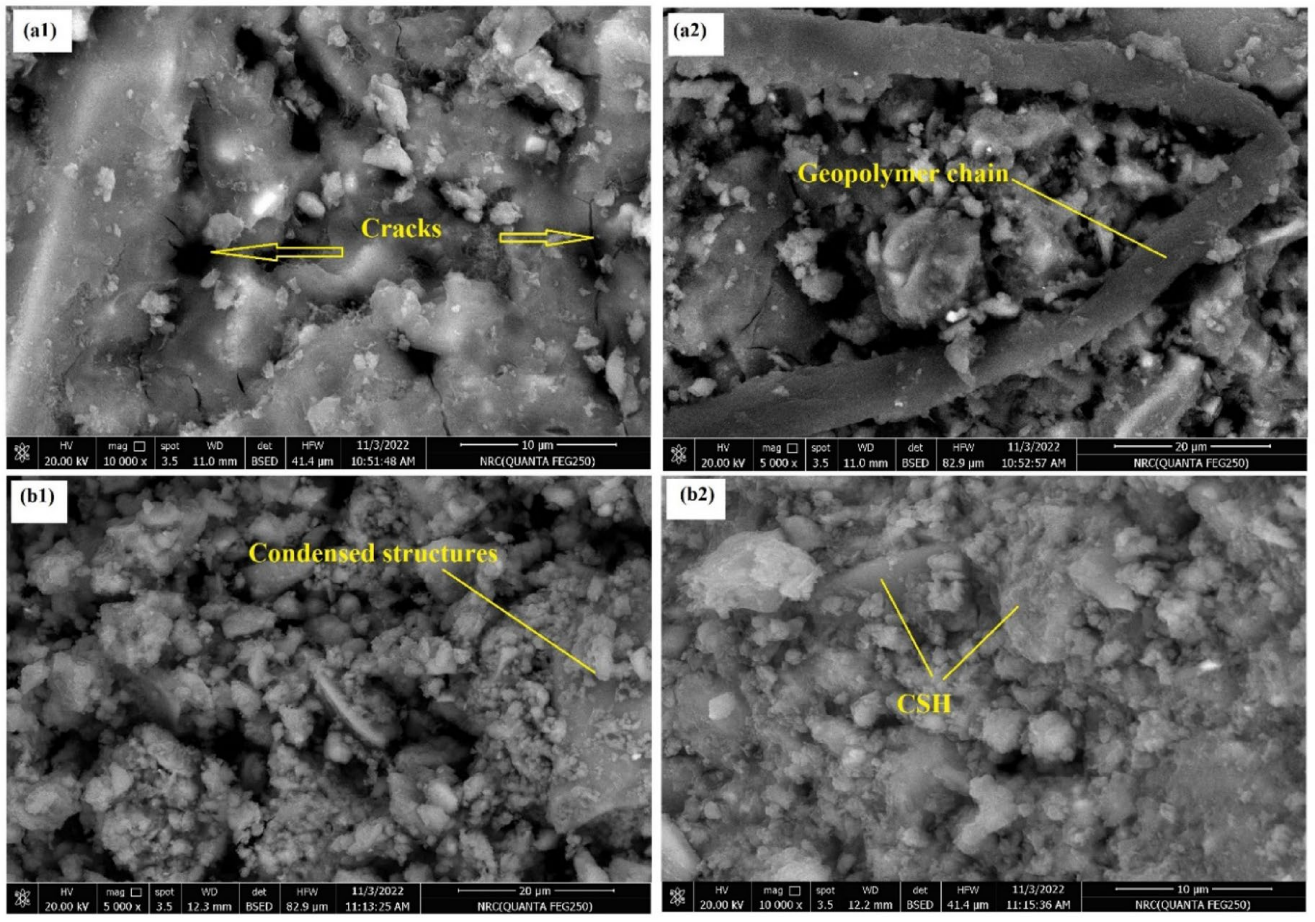
SEM images of the (**a1**, **a2**) M1, (**b1**, **b2**) M5 geopolymer pastes after 28 days of curing in 100% RH.

**Fig. 10:**
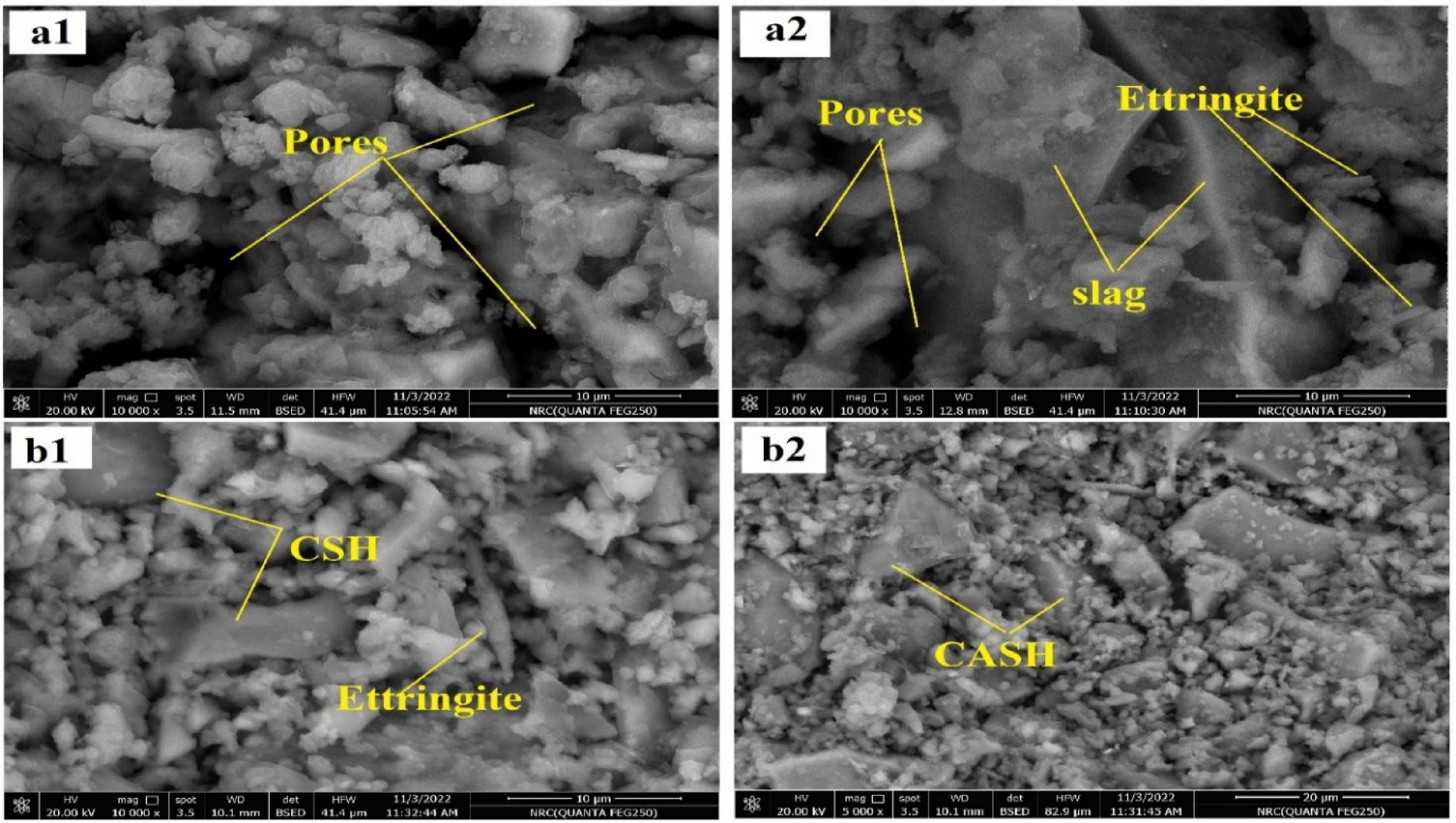
SEM images of the (**a1**, **a2**) M1, (**b1**, **b2**) M5 geopolymer pastes after immersion in MgSO_4_ solution for 3 months.

**Fig. 11:**
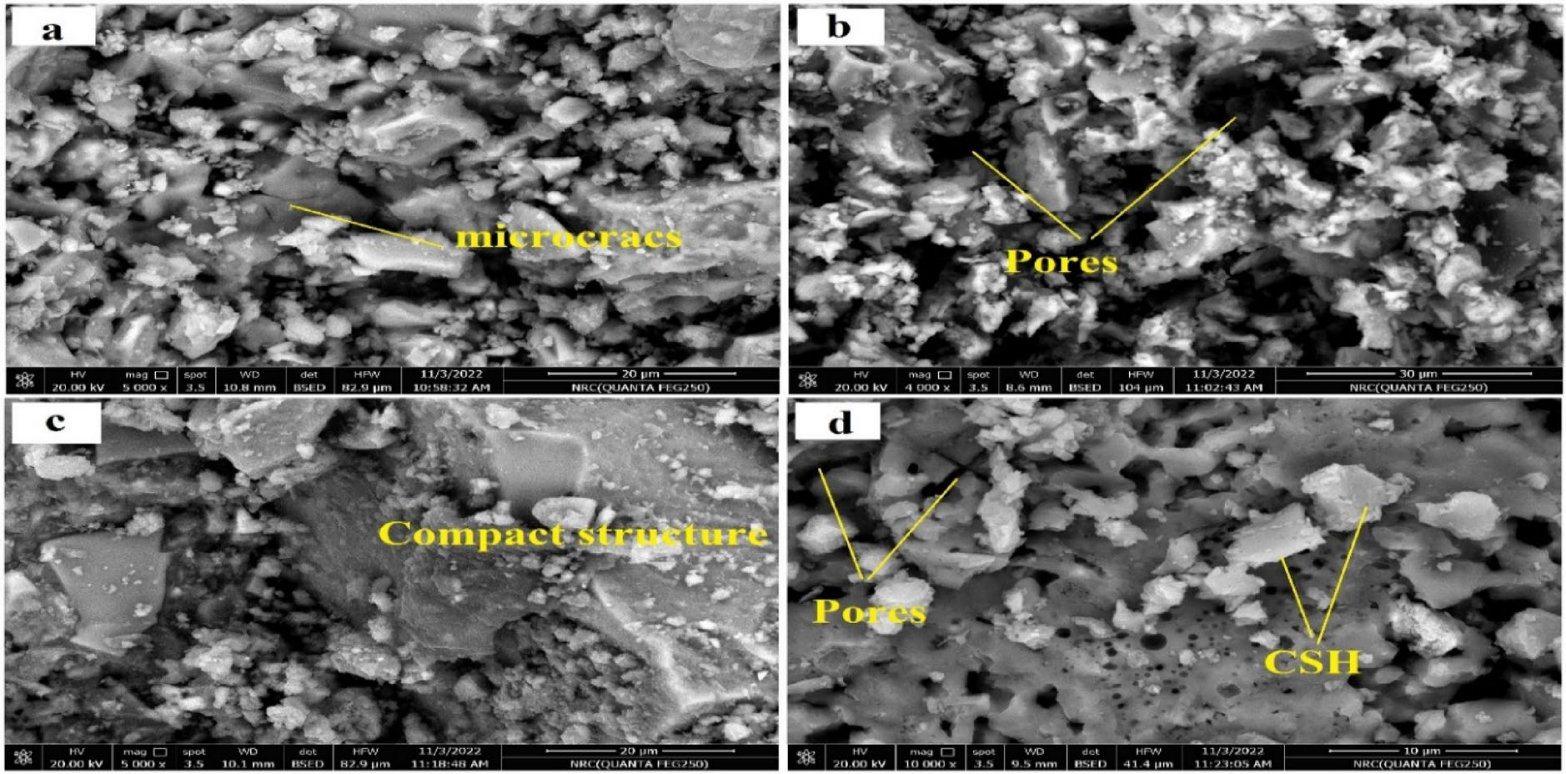
SEM images of (**a**, **b**) M1 after firing at 300 °C and 800 °C respectively, and (**c**, **d**) M5 after firing at 300 °C and 800 °C respectively.

Figures 9, 10 and 11 exhibit the SEM images of specimens M1 and M5 after curing for 28 days, immersion in MgSO_4_ solution for 3 months and firing at 300°,800 °C.

Figure 9 presents the SEM images of specimens M1 (a1, a2) and M5 (b1, b2) after 28 days of curing at 100% relative humidity. In both samples, SEM images reveal the formation of a dense, homogeneous gel-like matrix with embedded micro-fibrous or foil-like structures. These morphologies are typical of C–S–H and C–A–S–H gels in alkali-activated slag systems^69^. For Fig. 9a1,a2 it can be seen that the matrix structure is relatively loose and there are obvious cracks and interfaces compared to that appeared in Fig. 9b1,b2 which the gel network becomes denser and forms a condensed structure, indicating that the replacement of Na_2_SiO_3_ activator by Na_2_CO_3_ improves the compactness of the geopolymer matrix, contributes to optimizing the microstructure, and improves the mechanical properties of the geopolymer.

SEM images of M1 & M5 after immersion in MgSO_4_ solution for 3 months are illustrated in Fig. 10. In these images, needle-like or elongated crystalline structures are observed, which are characterized to Ettringite phase. These crystals are formed due to the interaction between the SO_4_^−2^ ions and the geopolymer hydrates. Such crystals, characterized by their large volumes, which upon formation inside the pores, cause an increase in internal pore pressure, which subsequently induces cracking^70^. That explains the depletion of the compressive strength observed for M1 mix due to its immersion in sulfate solution for 3 months. However, in Fig. 10a1,a2, limited crystals of Ettringite could be seen, and the matrix remains comparatively denser. This demonstrates the high resistance of M5 mix to the destructive effect of SO_4_^−2^ ions [71, 72].

SEM images of M1 and M5 samples after firing at 300 °C and 800 °C illustrated in Fig. 11a–d. SEM images in Fig. 11a,b showed formation of micro cracks and open pores, as well as crystals of anhydrous C_3_S phases, which appeared especially after firing at 800 °C [73].

For M5 mix, limited pores and cracks appear after 300 °and 800 °C Fig. 11c,d, compared to those for mix M1 indicted that M5 mix experienced gradual dehydration without severe structural collapse Such results explained the low mechanical properties and the failure in compressive strength for M1 than M5 after exposure to thermal treatment at 300 & 800 °C. This improved fire resistance of M5 mixes is attributed to a more polymerized, sodium-modified aluminosilicate gel network with lower carbonate residue and enhanced structural integrity under thermal stress.

## Conclusions

In this study, the slag system is activated to create GP cement using a blended activator consisting of either Na_2_SiO_3_ + CaCO_3_ or Na_2_SiO_3_ + Na_2_CO_3_. The following is a summary of the main conclusions of this study:

1. When activating the slag system with a combined activator of (SS + CC) or (SS + NC), the setting times are postponed compared to the mix activated with SS only. The drop in the GP matrix’s pH caused by substituting CC or NC for SS is linked to the setup delay. However, this delay in the setting is in acceptable value since it ranged between 138 min for the initial setting and 165 min for the final setting time.
2. The compressive strength was significantly decreased by 45 to 90% when the sodium silicate activator was partially substituted with calcium carbonate, as opposed to when sodium carbonate was employed exclusively. In contrast, replacement of SS by NC had improved the strength by 10–12%. This made NC is the suitable green activator that can replace SS effectively in GP mix.
3. M5 that’s contain (7% SS + 3% NC), shows the smallest (WA, %) and (TP, %), also, and exhibits the highest BD value by enhancing the geopolymerization reaction.
4. All geopolymer mixes prepared by the replacement of SS by CC exhibit a low resistance to sulphate solution, but replacement by NC shows higher resistivity with increasing replacement of 3% NC. Such results are attributed to the chemical attack of SO_4_^−2^ ions with the CC activator and/or the low geopolymer hydrates formed in such mixes activated by CC, in contrast to what happens if NC is applied.
5. Replacement of SS by NC shows better firing performance, especially by 3% replacement, which could retain about (70%, 51% and 39%) of their original strength before firing at 300, 600, and 800 °C respectively.
6. According to our findings, sodium carbonate can be used effectively with sodium silicate as an alkali activator, achieving acceptable strength and good durability in fire and sulfate environments.

Although this study demonstrates the technical feasibility of using Na₂CO₃ as a partial substitute for sodium silicate in one-part slag-based geopolymers, several limitations should be acknowledged. First, the investigation was limited to paste specimens; the behavior of corresponding mortar and concrete systems, including workability, shrinkage, and structural performance, was not examined. Second, the durability assessment focused primarily on sulfate attack and elevated temperature exposure, while other aggressive environments, such as carbonation, chloride penetration, freeze–thaw cycling, and long-term drying shrinkage, were not evaluated. Third, the study relied on XRD, FTIR, and SEM for phase and microstructural characterization; advanced quantitative techniques such as TGA/DTG, NMR, or MIP were not employed to fully elucidate reaction kinetics and pore structure evolution.

## Data Availability

Data will be made available upon request.
